# Switching and Torque Generation in Swarming *E. coli*

**DOI:** 10.3389/fmicb.2018.02197

**Published:** 2018-09-18

**Authors:** Katie M. Ford, Jyot D. Antani, Aravindh Nagarajan, Madeline M. Johnson, Pushkar P. Lele

**Affiliations:** Artie McFerrin Department of Chemical Engineering, Texas A&M University, College Station, TX, United States

**Keywords:** flagellar motor, motility, FliG, optical tweezers, chemotaxis

## Abstract

*Escherichia coli* swarm on semi-solid surfaces with the aid of flagella. It has been hypothesized that swarmer cells overcome the increased viscous drag near surfaces by developing higher flagellar thrust and by promoting surface wetness with the aid of a flagellar switch. The switch enables reversals between clockwise (CW) and counterclockwise (CCW) directions of rotation of the flagellar motor. Here, we measured the behavior of flagellar motors in swarmer cells. Results indicated that although the torque was similar to that in planktonic cells, the tendency to rotate CCW was higher in swarmer cells. This suggested that swarmers likely have a smaller pool of phosphorylated CheY. Results further indicated that the upregulation of the flagellin gene was not critical for flagellar thrust or swarming. Consistent with earlier reports, moisture added to the swarm surface restored swarming in a CCW-only mutant, but not in a FliG mutant that rotated motors CW-only (FliG^CW^). Fluorescence assays revealed that FliG^CW^ cells grown on agar surfaces carried fewer flagella than planktonic FliG^CW^ cells. The surface-dependent reduction in flagella correlated with a reduction in the number of putative flagellar preassemblies. These results hint toward a possibility that the conformational dynamics of switch proteins play a role in the proper assembly of flagellar complexes and flagellar export, thereby aiding bacterial swarming.

## Introduction

Swarming in flagellated bacteria is a type of surface-dependent motility that is marked by rapid and coordinated movements of groups of cells in coherent structures and swirling patterns on top of semi-solid surfaces (Henrichsen, 1972). Swarming has been implicated in several types of infections (Mobley and Belas, 1995; Callegan et al., 2006; Yang et al., 2017), as well as elevated antibiotic resistance (Kim and Surette, 2003; Kim et al., 2003; Overhage et al., 2008; Butler et al., 2010). The initiation of swarming is preceded by the arrival of planktonic cells on a surface – in a laboratory setting, this involves the inoculation of planktonic cells on agar substrates (Hughes and Kearns, 2017). Subsequently, the planktonic cells transition into a swarmer state (Belas, 2014). The swarmer state is often accompanied by, depending on the bacterial species, elongation of the cell body, expression of more number of flagella, multi-nucleation, and secretion of polysaccharides that promote surface motility (Harshey, 2003; Darnton et al., 2010; Kearns, 2010). In *Escherichia coli*, swarmer cells reportedly double their lengths but maintain similar flagellar numbers per unit surface area as the planktonic cells. Due to the proximity of the swarmer cells to a solid surface, the viscous drag experienced is likely high (Lauga et al., 2006; Zhang et al., 2009). It is unclear how cells overcome surface drag but one possibility is that motors in swarming bacteria adapt to produce higher torque than motors in planktonic cells (Tuson et al., 2013), although this remains untested. Another way that cells might overcome the drag is with the aid of the flagellar switch – it is known that the ability of the motor to switch its direction of rotation between clockwise (CW) and counterclockwise (CCW) is vital for swarming. Mutants that rotate motors CCW-only or CW-only fail to swarm whereas those that are able to switch their motors are able to swarm (Harshey and Matsuyama, 1994; Burkart et al., 1997). It has been proposed that switching aids in the lubrication of the surface by extracting water from the underlying agar, and possibly helps liberate cells from the secreted LPS (lipopolysaccharides), thereby facilitating cell movement (Wang et al., 2005; Mariconda et al., 2006). Much of this remains unexplained and these are unlikely to be the only mechanisms of drag reduction employed by swarming bacteria (Chen et al., 2007).

The flagellar motor rotates with the aid of stator units. Experiments in planktonic cells have shown that motors recruit stator units in greater numbers when the viscous loads are higher (Lele et al., 2013; Tipping et al., 2013; Terahara et al., 2017). Mechano-sensitive stator recruitment is one way to adapt torque in response to increased viscous drag near surfaces (Chawla et al., 2017). However, motor torque in swarming bacteria has never been measured. Torque enables the rotation of extracellular filaments, thereby resulting in a thrust on the cell body. Proper motor and filament assembly is, therefore, the key in coupling torque and cell propulsion. The initial steps in motor assembly include the formation of the FliG, FliM, and FliN complexes that form the flagellar switch (Schuster and Khan, 1994; Zhao et al., 1996; Macnab, 2003; Li and Sourjik, 2011). Concurrently, parts of the flagellar export apparatus begin to form with the final components, FliI, FliH, and FliJ, assembling prior to the formation of the flagellar hook (Kojima and Blair, 2004; Fukumura et al., 2017). The export complexes associate with FliN through the export apparatus component, FliH (McMurry et al., 2006; Paul et al., 2006; Minamino, 2018). Following the assembly of the rod and the flagellar hook, with a remarkable precision (Homma et al., 1990; Cohen et al., 2017), the anti-sigma factor FlgM is exported. This activates FliA, resulting in the transcription of class 3 genes including the flagellin protein, FliC, which forms the extracellular filament (Gillen and Hughes, 1991; Calvo and Kearns, 2015).

Reversals in the direction of rotation of the extracellular filament are mediated by the binding of an intracellular response regulator, CheY-P, to FliM and FliN, which promotes CW rotation in an otherwise CCW-rotary motor (Sarkar et al., 2010; Pandini et al., 2016). Biasing of the flagellar rotation in either the CCW or CW direction is known to cause remodeling of the flagellar switch complex, independent of CheY-P binding (Lele et al., 2012). The number of FliM and FliN subunits is fewer in motors that are locked in the CW direction and higher in the motors that are locked in the CCW direction (Paul et al., 2011b; Lele et al., 2012, 2015b; Branch et al., 2014; Delalez et al., 2014). Estimates indicate that there are numerous putative flagellar preassemblies in a planktonic cell (Sourjik and Berg, 2000; Delalez et al., 2010), although only three to four exhibit complete flagellar assembly. It is unclear if there is a role for the remodeling of FliM and FliN in swarming, however, deficiencies in the flagellar switch assembly do hamper filament formation (Konishi et al., 2009).

Here, we characterized torque generation and flagellar switching in swarmer cells of *E. coli.* Our measurements with wild-type *E. coli* indicated that the magnitude of torque generated in swarmer cells was similar to that in planktonic cells. However, the CW_bias_ (fraction of time that the motors rotate CW) was much lower in swarmer cells compared to that in planktonic cells. This reduction was dramatic, with ∼30% of the swarmer cells rotating CCW only. Despite the preference for CCW rotation in the swarmer state, the wild-type strain swarmed on agar substrates, whereas a CCW-only strain was unable. In agreement with earlier reports (Wang et al., 2005), the CCW-only strain could swarm in the presence of additional moisture on the agar surface. Experiments further indicated that transcriptional upregulation of the flagellin gene was neither critical for developing adequate flagellar thrust in the swarmer state nor for swarming. However, a CW-only mutant that rotates its motors exclusively CW due to a mutation in *fliG* (Togashi et al., 1997) could not swarm irrespective of the surface conditions. Using fluorescence visualization techniques, we found that the FliG^CW^ planktonic cells expressed fewer filaments compared to their wild-type planktonic counterparts. The number of FliG^CW^ filaments was even lower when the strain was grown on agar surfaces, which correlated with a reduction in the number of putative preassembled flagellar complexes. It is possible that flagellar assemblies in agar-grown cells are influenced, at least partially, by the conformations of the monomeric FliG subunits.

## Materials and Methods

### Bacterial Strains and Plasmids

Bacterial strains were derived from either RP437 or AW405 parent *E. coli* strains (**Table 1**). All plasmids were prepared with pTrc99A vector backbone, unless otherwise noted. Chromosomal alterations were achieved with the λ-red mediated homologous recombination technique (Datsenko and Wanner, 2000).

**Table 1:** Bacterial strains.

Strain/plasmid	Background	Genotype	Source
CCW strainccc5ptbbb180†	RP437	Δ*cheY*	This work
CW strainccc5ptbbb180†	RP437	Δ*cheRcheBcheZ*	This work
FliG^CW^ strainccc5ptbbb180†	RP437	*fliG^CW^*	Howard Berg lab
HCB1737 (wild type)^∗^	AW405	*fliC^Cys^*	Howard Berg lab
VSJ207 (FliG^CW^)^∗^	AW405	Δ*cheY*, *fliG^CW^*, *fliC^Cys^*	Howard Berg lab
FliG^CW^ *fliM-Y-fliM*	RP437	fliG^CW^, *fliM-eYFP(A206K)-fliM*	This work
*fliM-Y-fliM*, *CheY^∗∗^*	RP437	*fliM-eYFP(A206K)-fliM*, CheYD13KY106W	This work
HCB909	pXYZ202	CheYD13KY106W	Howard Berg Lab
pPL1	pTrc99A	*fliG^CW^*	Lele and Berg (2015)
pPL14	pTrc99A	*motAmotB*	Lele and Berg (2015)
pPL33	pTrc99A	*fliA*	This work
pPL40	pTrc99A	*fliF*	This work
pPL42	pTrc99A	*fliI*	This work
pPL47	pTrc99A	*flhA*	This work

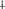
Corresponding strains were prepared to carry the *fliC^*sticky*^* allele to enable flagellar tethering and motor assays. ^∗^A cysteine replacement in the native *fliC* allele enabled fluorescent labeling of the flagella.

### Media

Overnight cultures were grown from isolated colonies in 5 mL of Tryptone Broth (TB) at 30 °C. Day cultures were grown by diluting 100 μL of overnight culture in 10 mL of fresh TB at 33°C to OD_600_∼0.5. Swarm-agar plates (Peptone, 10 g/L; NaCl, 5 g/L; beef extract, 3 g/L; 0.45% Eiken Agar, 0.5% Glucose) were prepared fresh, poured, and allowed to dry for 20 min open-faced on the work bench prior to inoculation with the strain of interest (2 μL, overnight culture grown at 30°C). Motility buffer (0.01 M phosphate buffer, 0.067 M NaCl, 10^-4^ M EDTA, 0.01 M sodium lactate and 1 μM methionine, pH∼7.0) was employed in motility assays.

### Motility Assays

#### Tethered Motors

Cells were prepared for tethered cell-assays by washing several times in motility buffer followed by shearing of the flagella, as described previously (Ford et al., 2017). Flagellar tethering to beads or coverslips was achieved with a sticky *fliC* mutation (Scharf et al., 1998). Cell rotation was imaged and recorded on a Nikon Eclipse Ti-E with a 20× phase objective or a Nikon Optiphot with a 40× phase objective at ∼60 fps with a CCD camera (DCC1545M-GL, Thorlabs Inc). Bead rotation was imaged on a Nikon Optiphot with a 60× phase objective coupled to a photomultiplier setup (Yuan et al., 2010; Ford et al., 2017).

#### Swarming

Swarm assays were carried out in an environmental chamber (ETS Model 5472, Electro-Tech Systems, Inc) that allowed for a precise control over humidity and temperature. Swarm plates were incubated for 8–10 h following inoculation at 75% relative humidity and 30°C, and then allowed to dry open faced for 2–4 h to increase the density of growth before imaging with a gel imager (ChemiDoc Touch Imaging System, BioRad). To add moisture to the agar surface, plates were sprayed with water from a spray bottle until the agar surface was visibly wet. The surface was allowed to dry with the lid open for 1 min before inoculation. The amount of water added in such an approach was 0.38 ± 0.03 mL. As an additional check, swarming was visually confirmed by observing colony movement within a region of the swarm plate with a 40× phase objective on a Nikon Optiphot.

#### Swimming

Planktonic cells were diluted in either fresh TB (1:10 dilution) or suspended in motility buffer. The dilute suspension was observed in a standard flow cell and cell motion was recorded away from either surface (coverslip or the microscope slide) at ∼60 fps with a CCD camera (DCC1545M-GL, Thorlabs Inc.) In the case of swarmer cells, cells were recovered from swarm plates by gently pouring 10 mL of motility buffer on the agar surface and swirling to dislodge surface-associated cells. Experiments were also done where cells were recovered specifically from the leading edge of the swarm and from the center of the colony; however, the CW_bias_ was similar in cells recovered from the two areas. The supernatant was collected in a Falcon tube and introduced in a flow cell for observation under a Nikon Optiphot microscope.

### Fluorescence Microscopy

A Nikon Ti-E microscope with a 100 mW, 514-nm laser (Cobalt Fandango) focused in the back focal plane of a 60× TIRF objective was used to generate evanescent fields. An Andor iXon DU897 camera was used for capturing TIRF (total internal reflection fluorescence) images while a CCD camera (DCC1545M-GL, Thorlabs Inc.) was used for capturing phase contrast images. Strains carrying *fliC*^cys^ were labeled with maleimide dye as described elsewhere (Blair et al., 2008). The *fliM-eYFP-fliM* internal fusion was gifted to us by the Berg lab. The allele carried a [Gly Gly][YFP_Ser_…YFP_Lys_][Ser Gly Gly] insertion between codons 15 and 16 of *fliM*. Tethered motor assays indicated that the fusion motors were fluorescent and functional.

### Motor Stall Assays

Optical traps were generated with a 976-nm laser (Azurlight ALS-IR-976-10-I-SF) by overfilling the back-aperture of a 60× objective. Optically trapped beads or cell bodies were then used to physically interrupt the rotation of tethered cells by moving the trapped object in the path of a rotating cell. The trap strength was adequate to prevent rotation of all tethered cells. When the traps were turned off, the cell body was free to rotate again.

### Isolation of RNA and Preparation of cDNA

Prior to extraction, bacterial strains were grown in fresh liquid swarm medium at 30°C or grown on solid swarm plates as previously described. All swarm assays for directionally biased cells were carried out by adding moisture and cells were only collected from plates that indicated successful swarming. Cell suspensions were centrifuged at 1000 *g* for 3 min, re-suspended in RNAlater^®^, and stored at 4°C for 1 day, after which time cells were transferred to -80°C for storage. RNA was extracted using an illustra RNAspin Mini Kit manufactured by GE Life Sciences. RNA was quantified using a NanoDrop 2000c (ThermoFisher Scientific) and a sample was run on a gel to check for ribosomal bands. The RNA was then converted into cDNA using qScript cDNA SuperMix manufactured by Quantabio. Separate reactions were carried out for the gene of interest –*fliC* (5′GCACCACCAGCATCGTT TGTAGTT3′) and the housekeeping gene –*gapA* (5′ACCGGTAGAAGACGGGATGATGTT3′).

### Real-Time PCR

A LightCycler^®^ 96 Real-Time PCR System (Roche) was employed for real-time PCR. Each reaction (20 μL) was carried out in a 96-well optical grade PCR plate, sealed with optical sealing tape. Amplifications were carried out using SYBR^®^ Green JumpStart^TM^ Taq ReadyMix^TM^ manufactured by Sigma-Aldrich, 2 μL of cDNA, and 250 nM of each primer (see primer information in **Supplementary Table S1**). Three biological and two technical replicates were carried out for each strain in planktonic and swarming cells. Relative quantification was performed by the ΔΔC_T_ method.

### Data Analysis

Videos of tethered cells were analyzed with custom-written codes in MATLAB to find the rotational speed as a function of time (Lele et al., 2015a). Time-averaged CW_bias_ values were determined for each tethered motor over the duration of 1–2 min. Mean speeds for cells were determined from Gaussian fits to speed distributions.

#### Swimming

Most cells swam in straight lines for limited time periods in the liquid medium. For each cell, the frames over which straight line motion was observed were averaged which resulted in a single image with bright streaks on a gray background. The corresponding length of the straight line intensity profile was determined and divided by the period of observation to obtain swimming speed.

### Statistical Analysis

All statistical analyses were performed with the Student’s *T*-test. Results with *p* < 0.05 were considered statistically significant.

## Results

### Flagellar Switching in Swarmer Cells

**FIGURE 1:**
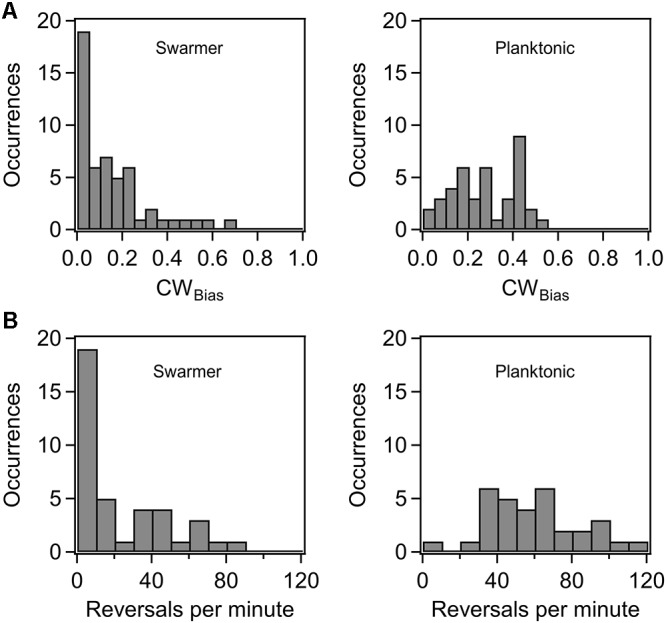
**(A)** Probability of CW rotation in wild-type swarmers and planktonic cells. The mean CW_Bias_ was 0.27 ± 0.02 (*n* = 40 motors) for the wild-type planktonic cells and 0.15 ± 0.02 (*n* = 52 motors) for the wild-type swarmer cells. The difference in the mean CW_Bias_ for planktonic and swarmer cells was significant (*p* < 0.05). **(B)** Distribution of motor reversals observed in wild-type swarmer and planktonic cells. The mean reversal rates were 22.4 and 59.9 reversals/min, respectively. The difference in the means was significant (*p* < 0.05).

Previous research indicated that the flagellar switch is able to adapt to mechanical stimuli, although the mechanisms are presently unknown (Fahrner et al., 2003; Lele et al., 2013). To determine how cells adapt flagellar motor functions in order to continue their surface existence, we characterized motor behavior in swarmer cells. We inoculated overnight cultures of a wild-type strain (RP437 background) that carried a sticky *fliC* allele on standard swarm agar plates. The colonies swarmed and were recovered from the plates several hours later, as discussed in Section “Materials and Methods.” The swarmer cells were then sheared and washed in motility buffer, before tethering to glass surfaces. The CW_Bias_ (fraction of time that motors rotate CW) was quantitatively determined from digital recordings of cell rotation with custom-written MATLAB codes (Lele and Berg, 2015). Reversal frequencies were also determined. As shown in **Figure 1A**, the average CW_bias_ in swarmer cells (left panel) was lower than that in the corresponding planktonic cells (right panel). The distribution in the case of the former was skewed toward a CW_bias_ ∼0, with 36% of cells rotating CCW only. The reversal rates further illustrated this disparity. **Figure 1B** indicates that the reversal rates were lower in the case of the swarmer cells (left panel) compared to those observed in the planktonic cells (right panel). These observations are consistent with a recent work that observed lower tumbling frequencies in swarmer cells in comparison to planktonic cells grown in a liquid medium (Turner et al., 2016). This result is unexpected considering that switching of the flagella has been reported to be crucial for swarming (Burkart et al., 1997). The lower CW_bias_ likely indicates that the pool of phosphorylated CheY may be reduced in swarmer cells in comparison to planktonic cells, although differential acetylation levels could also play a role (Liarzi et al., 2010).

To test the role of the flagellar switch in swarming, several directionally biased mutant strains were constructed from the parent AW405 and RP437 wild-type strains. In general, two types of strains were employed in swarm-experiments: a strain lacking *cheY* in which motors rotated exclusively CCW (*CCW strain*) and a *fliG* mutant strain in which motors rotated exclusively CW (*FliG^CW^ strain*) because the FliG subunits were locked in the CW conformation (Togashi et al., 1997; Lele and Berg, 2015). In addition, a strain lacking *cheR-cheB-cheZ* was also constructed in which motors rotated predominantly CW (*CW strain*) due to an excess of [CheY-P] (Sourjik and Berg, 2002). As anticipated, all the directionally biased mutants failed to swarm in a standard swarm assay, unlike the wild-type strains. Swarming was restored in the CCW strain when CheY was expressed from an inducible plasmid. In agreement with earlier reports (Wang et al., 2005), it was possible to restore swarming, at least partially, in the CCW and CW strains by moistening the agar surface with water (see Section “Materials and Methods”; **Supplementary Figure S1**). Next, the CCW strain was transformed with an inducible vector from which a constitutively active form of CheY (CheYD13KY106W) was expressed (Scharf et al., 1998). Background expression was adequate to predispose tethered cells in this strain to rotate CW-only. It was possible to restore swarming partially in this strain by adding moisture. Conversely, swarming could not be restored in the FliG^CW^ strain. These results suggested that the ability to swarm was not completely inhibited by switch inactivation in strains that carried the wild-type *fliG* allele. However, the CW conformation of the monomeric FliG subunits in the FliG^CW^ strain probably precluded swarming.

### Torque and Swarming

**FIGURE 2:**
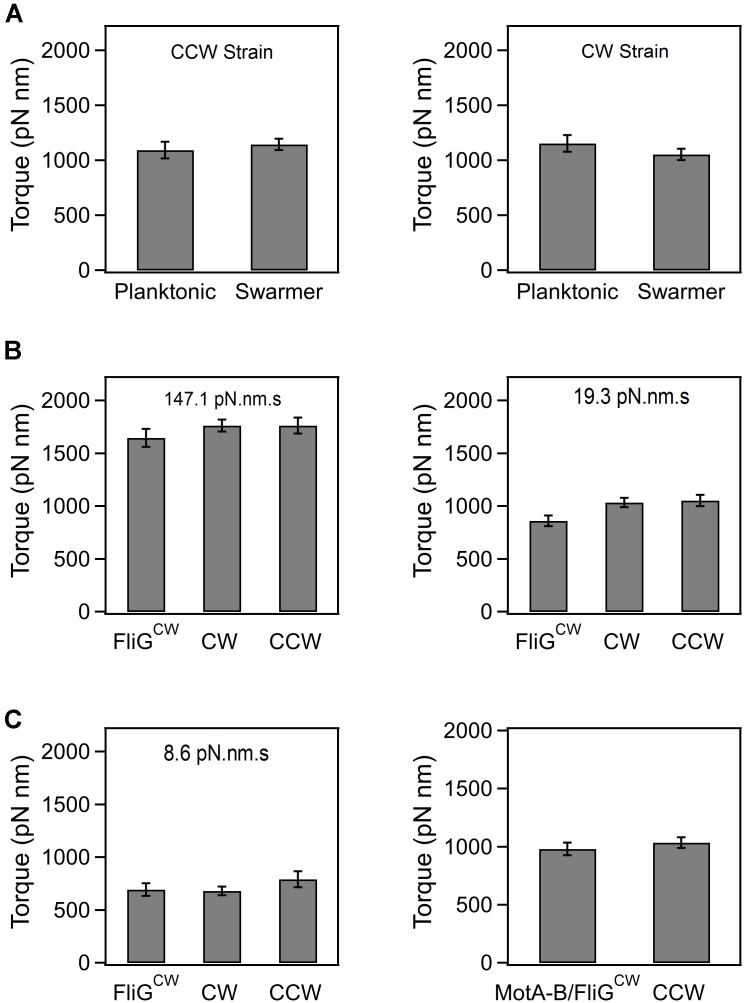
**(A)** Average torque generated in planktonic and swarming cells belonging to the CCW and CW strains. The average torque was 1154.9 ± 75.4 pN⋅nm (*n* = 35 motors, swarmer CCW strain) and 1121.7 ± 57.9 pN⋅nm (*n* = 32 motors, planktonic CCW strain). Differences in the mean torques were not significant (*p* > 0.05). The average torque was 1093 ± 81.5 pN⋅nm (*n* = 35 motors, swarmer CW strain) and 1144.5 ± 129.1 pN⋅nm (*n* = 16 motors, planktonic CW strain). Differences in the mean torques were not significant (*p* > 0.05). **(B)** Torque generated by the FliG^CW^ strain in comparison to that in CCW and CW strains. At high loads (147.1 pN**⋅**nm**⋅**s), the differences in means were statistically insignificant. At medium loads (19.3 pN**⋅**nm**⋅**s), the differences in the mean torque in the FliG^CW^ strain and the other strains were significant. At lower loads (8.6 pN**⋅**nm**⋅**s), the difference in the mean torque in the FliG^CW^ strain and the CCW strain was significant. **(C)** The slight degradation in the torque in the FliG^CW^ strain seen in **(B)** was remedied by expressing extra copies of MotA and MotB subunits in the strain from an inducible plasmid. The difference in mean torques in this strain and the CCW strain was insignificant at a load of 19.3 pN**⋅**nm**⋅**s. All plots indicate standard error.

In order to determine if the loss in swarming ability in the directionally biased mutants was, in part, due to an emergent deficiency in torque generation following surface inoculation, we measured the speed of rotation in tethered cells that had been recovered from agar substrates. Based on the size of the cells and the mean speeds, we calculated the average torque generated in the CCW and CW strains (see Section “Materials and Methods”). As indicated in **Figure 2A**, there were no significant differences in the mean torques generated in the planktonic and the agar-grown cells, irrespective of whether they belonged to the CCW or CW strain. The same was true in the case of the wild-type planktonic and swarmer cells (data not shown). In the FliG^CW^ strain, we measured the mean torque over a range of viscous loads. The viscous load was varied by employing latex beads of different sizes (2, 1, and 0.75 μm) and rotation rates were measured via a photomultiplier-based high speed tracking technique (Yuan et al., 2010; Ford et al., 2017). Torque was calculated from rotation speeds and bead sizes (Ryu et al., 2000). As shown in **Figure 2B**, the differences between the mean torque generated by the FliG^CW^ motors and that developed by the CW and CCW motors were statistically insignificant at high loads (load = 147.10 pN⋅nm⋅s). At lower loads (19.31 pN⋅nm⋅s), these differences were significant – FliG^CW^ motors produced ∼15% less torque than the CW motors. At the lowest loads, we employed (8.55 pN⋅nm⋅s), the difference between the average torque in FliG^CW^ motors and that in the CW strain was again insignificant, whereas that between the FliG^CW^ motors and the CCW strain was significant, as expected from the anisotropy in torque generation in the two directions of motor rotation (Yuan et al., 2010; Lele et al., 2015b). To assess if the complete inhibition of swarming in the FliG^CW^ strains arose due to such minor variations in torque, we transformed the FliG^CW^ strain with an inducible plasmid carrying the *motAmotB* genes. Higher expression of MotA–MotB levels increased the average torque in the FliG^CW^ strain to a level that was similar to that in the CCW strain (load = 19.31 pN⋅nm⋅s, **Figure 2C**). Yet, the same level of induction failed to restore swarming in the FliG^CW^ strain. Together, the results indicated that swarmers are unlikely to develop higher flagellar power compared to planktonic cells at a given viscous load, in order to compensate for the increased surface drag.

### Flagellar Thrust in Agar-Grown Cells

Although our data suggests that differential torque generation is not the reason for the loss of swarming in directionally biased mutants, a reduction in flagellar thrust could play a role. Flagellar thrust depends on a rich interplay between the polymorphic form, arc lengths of the filament, filament numbers, and the ability to form tight flagellar bundles. Rather than making independent measurements of each of these factors, we opted to measure swimming speeds of the cells. The speeds encompass each of the key factors that influence motility, including cell lengths, enabling comparisons between different types of cells. To do this, swimming speeds were measured in the planktonic and agar-grown cultures in all the aforementioned strains. Agar-grown cells were recovered by adding and gently swirling motility buffer on the swarm substrates. Motility was subsequently recorded in standard flow cells and quantitatively analyzed; the data are shown in **Table 2**. Speeds measured in strains that were able to swarm on agar and were able to swim in motility buffer have been labeled as “*swarmer speeds*.” Speeds measured in strains that did not swarm but were able to swim in motility buffer have been labeled as “*non-swarmer speeds*.” Speeds measured in strains that were grown in liquid media have been labeled “*planktonic speeds*.” There was no significant difference in the mean speeds measured in planktonic cells belonging to the directionally biased strains carrying the native *fliG* allele and the wild-type cells. However, the difference in the mean speeds in planktonic cells belonging to the FliG^CW^ and the wild-type strains was statistically significant; FliG^CW^ swam at ∼33% lower speed. This indicated degradation in the flagellar thrust in FliG^CW^ planktonic cells. In the case of cells recovered from the agar surfaces, the differences between the mean speeds in FliG^CW^ and the wild type were further amplified. The wild-type swarmer cells experienced an increased flagellar thrust by ∼ 9% relative to the wild-type planktonic cells. By contrast, the agar-grown FliG^CW^ cells were mostly non-motile. Among the ones that exhibited motility, the average swimming speed was ∼1/2 the speed of the wild-type swarmers. In comparison, CCW and wild-type swarmer speeds were similar but the CCW non-swarmer speed was ∼20% lower than the wild-type swarmer speed. This suggested that the inhibition of swarming in the CCW mutant (in the absence of added moisture) was partially attributable to reduced flagellar thrust. Considering that the flagellar motor torque is not deficient, this indicated a degradation in either the flagellar lengths or the number of flagella in the directionally biased mutant. In the case of FliG^CW^, this deficiency was extreme. In the wild type, there was no significant difference in the swimming speeds of swarmers in the presence or absence of added moisture on the agar surface.

**Table 2:** Mean swimming speeds for planktonic and agar-grown cells.

Strain name	Planktonic	Non-swarmer	Swarmer
Wild type	24.5 ± 0.5 (*n* = 40)	**–**	26.7 ± 1.0 (*n* = 30)
CCW	22.4 ± 0.5 (*n* = 30)	21.7 ± 1.0 (*n* = 30)	26.4 ± 0.8 (*n* = 30)
fliG^CW^	16.5 ± 0.6 (*n* = 30)	15.3 ± 0.6 (*n* = 30)	**–**

### Loss of Swarming in FliG^CW^ Mutant

#### Filament Numbers

**FIGURE 3:**
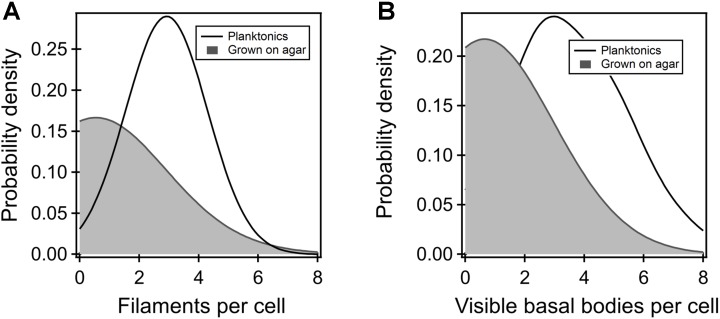
**(A)** Kernel density estimates of the flagellar filaments per cell in the planktonic (black curve) and agar-grown FliG^CW^ strain (gray-shaded region). The difference in means was significant. Filaments were not observed in more than 57% of the agar-grown FliG^CW^ cells. The average filament number observed in the planktonic cells was lower than that observed in wild-type planktonic cells (4 ± 1). **(B)** The number of putative flagellar preassemblies were determined from TIRF measurements. There were fewer such bodies in the agar-grown FliG^CW^ cells (number of preassemblies = 0.64 ± 0.16, *n* = 28 cells) relative to the planktonic FliG^CW^ cells (number of preassemblies = 3.72 ± 0.27, *n* = 29 cells), as seen from the kernel density estimates from the raw data. The difference in the means was significant (*p* < 0.05).

To determine why the flagellar thrust in the FliG^CW^ strain was dramatically lower in comparison to other strains, we employed fluorescence visualization techniques and measured the number of filaments in planktonic as well as agar-grown cells. Filaments carrying cysteine residues were labeled with a maleimide-based fluorescent dye and visualized as detailed in Section “Materials and Methods.” The filament numbers were manually counted from the fluorescence microscopy images. The average number of filaments per cell in the planktonic FliG^CW^ strain was determined to be 3 ± 1 (**Figure 3A**). The average number in the planktonic wild-type cells was 4 ± 1 (not shown). Thus, there was ∼25% drop in the number of flagella per cell in the planktonic FliG^CW^ cells. This was consistent with a 33% decrease in swimming speeds in the planktonic FliG^CW^ cells. In comparison, the distribution of the number of filaments per cell in the agar-grown FliG^CW^ strain was skewed toward zero (**Figure 3A**). Of the 200 cells analyzed, 115 cells (57.5%) appeared to carry no filaments. In the cells that had visible flagella, the average number of filaments per cell was 1.6 ± 0.1. In wild-type swarmer cells, only 12 cells out of 115 appeared to carry no filaments. In the cells that had visible flagella, the average number of filaments per cell was 3.0 ± 0.2 (see **Supplementary Figure S2**), in agreement with recent measurements of wild-type *E. coli* swarmers by Turner et al. (2016). The reduction in the number of visible filaments in the FliG^CW^ cells was correlated with the complete loss of swarming ability. Additionally, upon visual inspection of these filaments, filaments appeared to be shorter than those found in wild-type cells.

#### Flagellar Susceptibility to Shear

One explanation for the lower filament numbers in the FliG^CW^ strain, planktonic or otherwise, could be that the flagellum is more susceptible to shear, similar to the reported propensity of the flagella to shear in *fliL* mutants of *Salmonella enterica* (Attmannspacher et al., 2008). To test this hypothesis, we employed optical traps to stall tethered motors of the FliG^CW^ strain. Stalling ensures that the flagellar motor delivers the maximum possible force on the rotor (Ryu et al., 2000), thereby subjecting the flagellum to high physiologically relevant shearing forces. Several tethered motors were stalled for approximately 10 min (*n* = 10 cells), as described in Section “Materials and Methods.” Shearing of the flagellum was expected to be detectable through the detachment of the cell from the surface during stalling or through the loss of the ability to rotate following the removal of the optically trapped bodies. In all of the experiments, flagellar motors in the FliG^CW^ strain remained tethered and functional even after trap removal. This indicated that the FliG mutation is unlikely to result in flagella that are easily sheared under high viscous loads.

#### Intracellular Protein Levels

We tested whether incomplete or inefficient assembly of the export apparatus could be compensated for by increasing the expression levels of the export ATPase, FliI, or the levels of FlhA. The latter is a part of the export gate of the type 3 secretion system and forms a dock for FliI (Abrusci et al., 2012). FliI overexpression especially has been shown to partially restore flagellation in strains carrying partial deletions in the switch proteins (Konishi et al., 2009; Erhardt and Hughes, 2010). However, overexpression of these two proteins from inducible plasmids failed to restore swarming in the FliG^CW^ strain (**Supplementary Figure S3**). Similarly, overexpression of FliF and FliG^CW^ also failed to elicit swarming in that strain.

#### Flagellin Regulation

The reduced flagellar thrust and filament numbers could arise due to a reduction in the flagellin levels in the directionally biased mutants. To test this, qPCR experiments were performed as detailed in Section “Materials and Methods.” Briefly, the flagellin gene was selected based on earlier reports that *fliC* was one of the only genes that is differentially regulated in wild-type swarmers in *Salmonella typhimurium* (Wang et al., 2004). Comparisons were made between the transcriptional levels of *fliC* in the planktonic and swarmer cell types for each of the three strains: the wild-type, the CCW, and the FliG^CW^ strain. In the case of the CCW and the FliG^CW^ strain, the cells were recovered from agar substrates that had been treated with water to increase moisture. Swarming was observed in the former but not in the latter strain. FliC mRNA levels were upregulated by twofold in wild-type swarmers, but there was no significant change in the two directionally biased strains (**Supplementary Table S2**). It was interesting to note that although the flagellin gene was not significantly upregulated in the agar-grown CCW cells, unlike the wild-type, swarming was not inhibited. Flagellar thrust was not diminished in CCW swarmers either. We did not observe a downregulation in flagellin transcription in the agar-grown FliG^CW^ cells (relative to the planktonic cells) despite a clear reduction in the number of filaments. These observations were further supported by swarm experiments where *flgM* was deleted in the FliG^CW^ strain. The anti-sigma factor, FlgM, binds to FliA and prevents the transcription of class 3 genes. Inefficient functioning of the export apparatus could result in decreased FlgM export, preventing the transcription of flagellar genes. The Δ*flgM fliG^CW^* strain, however, failed to swarm; a wild-type strain deleted for *flgM* retained its swarming ability. Prior observations also suggest that the deletion of *flgM* in a CCW strain did not restore swarming (Wang et al., 2005). Thus, a reduction in the expression of flagellar genes due to the inactivation of FliA by un-exported FlgM is unlikely to be the reason for the loss of swarming in the directionally biased mutants. Instead, the data pointed to inefficient flagellar assembly as the key problem.

### Flagellar Preassemblies

#### FliM Assembly in Planktonic Cells

Previous *in vivo* TIRF measurements with fluorescent fusions of FliM indicate that several putative flagellar preassemblies can be found throughout the cell membrane in planktonic cells of *E. coli*, although only three to four exist as complete flagella (Sourjik and Berg, 2000; Delalez et al., 2010). These assemblies are identifiable as fluorescent foci (Li and Sourjik, 2011). In each functional motor, there are 34–45 molecules of FliM subunits (Park et al., 2006; Delalez et al., 2010; Paul et al., 2011a; Lele et al., 2012). Remodeling of FliM is dependent on the direction of rotation of the motor and not on the interactions with CheY-P *per se* (Lele et al., 2012, 2015b). The assembly of FliN is proportionate to that of FliM (Branch et al., 2014; Delalez et al., 2014). The assembly of FliM is not disrupted in the FliG^CW^ mutant; previous observations indicate that the number of FliM subunits per motor in the FliG^CW^ strain is quantitatively similar to that observed in wild-type motors that rotate CW-only due to an excess pool of phosphorylated CheY (Lele et al., 2012; **Supplementary Figure S4**). Furthermore, incomplete or deficient FliM assembly is known to degrade motor torque (Tang and Blair, 1995), whereas our torque measurements (**Figures 2B,C**) indicate little or no degradation in torque in FliG^CW^ motors, over the range of viscous loads studied here. It is likely, then, that the reduction in the number of filaments in planktonic cells of the FliG^CW^ strain, relative to the wild-type, is not due to a defective C-ring assembly. Rather, the reduction in filaments could be due to subtle changes in the interactions of the export apparatus with FliG–FliM–FliN protomers that have adopted a locked CW conformation.

#### FliM Assembly in Cells Grown on Agar

Next, we attempted to determine whether the dramatic reduction in the number of filaments in FliG^CW^ cells grown on agar surfaces, relative to the planktonic FliG^CW^ cells, was entirely due to deficient flagellar export or whether the locked conformations of the FliG monomers inhibited the assembly of putative flagellar complexes on agar substrates. For this purpose, a FliG^CW^ strain was constructed that carried a genomic *fliM-eYFP-fliM* allele (Section “Materials and Methods”). Motors in this strain were functional and rotated CW-only when tethered. The strain was grown in liquid media and on agar surfaces. TIRF visualization enabled quantitative determination of the number of putative flagellar complexes with custom-written MATLAB codes for fluorescent foci detection (Lele et al., 2012). The distribution of the number of foci detected per cell is indicated in **Figure 3B** for the planktonic and agar-grown FliG^CW^ cells. The average numbers of foci observed here are fewer than those reported earlier (Delalez et al., 2010). This is likely because the TIRF field employed only allows visualization of ∼1/6th of the total volume of the cell body. As can be seen, there were less than half the number of foci in the agar-grown cells (*n* = 28 cells) when compared with the planktonic cells (*n* = 29 cells). This is consistent with our observations of very few filaments in the agar-grown FliG^CW^ cells, and the loss of swarming in this strain. To further test this notion, we attempted to construct the corresponding wild-type control for fluorescence assays. However, that strain was unable to swarm and as a result, was not employed in further experimentation. Nonetheless, to test whether the FliG^CW^ mutation, rather than the CW-locked conformation of the FliG monomers, was responsible for the reduction in the foci on agar, we constructed and tested a strain that carried the native *fliG* and the *fliM-eYFP-fliM* alleles on its chromosome, and an excess of the constitutively active CheY variant. Motors in this strain rotated CW-only and the strain did not swarm. A similar reduction in the number of foci in agar-grown cells was observed in comparison to the corresponding planktonic cells (**Supplementary Figure S5**). This suggested that it is not the FliG^CW^ mutation *per se* that interfered with the assembly of the putative flagellar assemblies in cells grown on agar surfaces, but possibly the lack of conformational transitions in the switch protomers. These experiments provide a measure of support to the notions that in cells that fail to swarm on agar, the number of putative flagellar preassemblies is decreased relative to that in the planktonic cells, and that the export of flagellar proteins is influenced by the conformations of the switch protomers.

## Discussion

Our results suggest that the flagellar motors in swarming cells of *E. coli* do not develop a higher power relative to the planktonic motors, for a given viscous load. This is in contrast to species such as *Pseudomonas aeruginosa*, where cells employ a specialized set of stator proteins that are capable of developing higher power when the cell finds itself near a surface (Kuchma et al., 2015). Unexpectedly, the CW_bias_ was lower in swarmer cells compared to that in planktonic cells, in fact, one out of three swarmer cells showed no inclination to reverse the direction of flagellar rotation. This is likely due to a smaller pool of phosphorylated CheY in the swarmer cells. This may be a consequence of an overall reduction in the chemotaxis protein abundances, or a reduction in the sensitivity of the flagellar switch. In any scenario, the reduction in reversal rates and CW_bias_ is at odds with the swarm assays that clearly emphasize the role of flagellar switching in swarming. We propose, then, that it is not switching *per se*, but rather some associated property of the wild type that enables swarming.

Our experiments also indicate that the transcriptional upregulation of the flagellin gene was neither critical for developing adequate flagellar thrust in the swarmer state nor for swarming. This conclusion was derived from the observation that the CCW strain did not upregulate the expression of the flagellin gene, but was still able to swarm, provided that moisture was added. Swarmer cells belonging to the CCW strain were also able to generate similar flagellar thrusts as the wild-type swarmer cells. This suggested that the upregulation of *fliC* expression might simply be a consequence of agar-based growth of the wild-type strain. Transcriptional activity was also unchanged between the planktonic and swarmer cells belonging to the FliG^CW^ strain. The deletion of *flgM* did not restore swarming in this strain. Considering that this strain was severely deficient in producing flagella on the agar substrate, this further suggested that the extreme defects in flagellar production on agar were unlikely due to reduced *fliC* expression. Optical tweezer experiments ruled out the possibility that the reduction in flagellar numbers in the FliG^CW^ strain was due to shearing near the agar surface. The *fliG^CW^* mutation in planktonic cells of *E. coli* did not interfere with the assembly and functioning of the flagellar C-rings, as evidenced by previous measurements of FliM assembly in a FliG^CW^ strain (Lele et al., 2012), and the torque measurements in the present work (**Figures 2B,C**). This suggested that the CW-locked conformation of the FliG–FliM–FliN protomers, rather than assembly defects, affected flagellar export in this strain. Finally, we also found evidence that the assembly of putative FliM assemblies in the FliG^CW^ cells was inhibited on agar surfaces, relative to the planktonic state. This decrease correlated with reduced flagellar numbers on agar surfaces, which was most likely responsible for the degradation in flagellar thrust and the loss of swarming. However, the corresponding wild-type fusion strain failed to swarm, which prevented us from determining if a high density of preassemblies was maintained in wild-type swarmers. Based on the available data, we speculate that flagellin export depends on the switch-activity, and not just assembly, and that the stochastic transitions in the conformations of switch protomers likely help anneal putative preassemblies. Under the conditions of increased shear near agar surfaces, locked protomeric conformations might result in inadequate assembly and inefficient export of flagellar substrates.

## Author Contributions

KF, JA, AN, and MJ performed the experiments and analyses. KF, AN, and PL developed the experimental setups and designed the research. KF, JA, and PL wrote the manuscript. All authors reviewed the manuscript.

## Conflict of Interest Statement

The authors declare that the research was conducted in the absence of any commercial or financial relationships that could be construed as a potential conflict of interest.

## References

[B1] Abrusci P. Vergara-Irigaray M. Johnson S. Beeby M. D. Hendrixson D. R. Roversi P. (2012). Architecture of the major component of the type III secretion system export apparatus. *Nat. Struct. Mol. Biol.* 20 99–104. 10.1038/nsmb.2452 23222644 PMC3537844

[B2] Attmannspacher U. Scharf B. E. Harshey R. M. (2008). FliL is essential for swarming: motor rotation in absence of FliL fractures the flagellar rod in swarmer cells of *Salmonella enterica*. *Mol*x. *Microbiol.* 68 328–341. 10.1111/j.1365-2958.2008.06170.284590 18284590

[B3] Belas R. (2014). Biofilms, flagella, and mechanosensing of surfaces by bacteria. *Trends Microbiol.* 22 517–527. 10.1016/j.tim.2014.05.002 24894628

[B4] Blair K. M. Turner L. Winkelman J. T. Berg H. C. Kearns D. B. (2008). A molecular clutch disables flagella in the *Bacillus subtilis* biofilm. *Science* 320 1636–1638. 10.1126/science.1157877 18566286

[B5] Branch R. W. Sayegh M. N. Shen C. Nathan V. S. J. Berg H. C. (2014). Adaptive remodelling by FliN in the bacterial rotary motor. *J. Mol. Biol.* 426 3314–3324. 10.1016/j.jmb.2014.07.009 25046382 PMC4150818

[B6] Burkart M. Toguchi A. Harshey R. M. (1997). The chemotaxis system, but not chemotaxis, is essential for swarming motility in *Escherichia coli*. *Proc. Natl. Acad. Sci. U.S.A.* 95 2568–2573. 10.1073/pnas.95.5.2568 PMC19416 9482927

[B7] Butler M. T. Wang Q. Harshey R. M. (2010). Cell density and mobility protect swarming bacteria against antibiotics. *Proc. Natl. Acad. Sci. U.S.A.* 107 3776–3781. 10.1073/pnas.0910934107 20133590 PMC2840483

[B8] Callegan M. C. Novosad B. D. Ramirez R. Ghelardi E. Senesi S. (2006). Role of swarming migration in the pathogenesis of *Bacillus* Endophthalmitis. *Invest. Ophthalmol. Vis. Sci.* 47 4461–4467. 10.1167/iovs.06-0301 17003440

[B9] Calvo R. A. Kearns D. B. (2015). FlgM Is secreted by the flagellar export apparatus in *Bacillus subtilis*. *J. Bacteriol.* 197 81–91. 10.1128/JB.02324-14 25313396 PMC4288692

[B10] Chawla R. Ford K. M. Lele P. P. (2017). Torque, but not FliL, regulates mechanosensitive flagellar motor-function. *Sci. Rep.* 7:5565. 10.1038/s41598-017-05521-8 28717192 PMC5514156

[B11] Chen B. G. Turner L. Berg H. C. (2007). The wetting agent required for swarming in *Salmonella enterica* serovar typhimurium is not a surfactant. *J. Bacteriol.* 189 8750–8753. 10.1128/JB.01109-07 17905988 PMC2168935

[B12] Cohen E. J. Ferreira J. L. Ladinsky M. S. Beeby M. Hughes K. T. (2017). Nanoscale-length control of the flagellar driveshaft requires hitting the tethered outer membrane. *Science* 356 197–200. 10.1126/science.aam6512 28408605 PMC5963725

[B13] Darnton N. C. Turner L. Rojevsky S. Berg H. C. (2010). Dynamics of bacterial swarming. *Biophys. J.* 98 2082–2090. 10.1016/j.bpj.2010.01.053 20483315 PMC2872219

[B14] Datsenko K. A. Wanner B. L. (2000). One-step inactivation of chromosomal genes in *Escherichia coli* K-12 using PCR products. *Proc. Natl. Acad. Sci. U.S.A.* 97 6640–6645. 10.1073/pnas.120163297 10829079 PMC18686

[B15] Delalez N. J. Berry R. M. Armitage J. P. (2014). Stoichiometry and turnover of the bacterial flagellar switch protein FliN. *mBio* 5:e01216-14. 10.1128/mBio.01216-14 24987089 PMC4161238

[B16] Delalez N. J. Wadhams G. H. Rosser G. Xue Q. A. Brown M. T. Dobbie I. M. (2010). Signal-dependent turnover of the bacterial flagellar switch protein FliM. *Proc. Natl. Acad. Sci. U.S.A.* 107 11347–11351. 10.1073/pnas.1000284107 20498085 PMC2895113

[B17] Erhardt M. Hughes K. T. (2010). C-ring requirement in flagellar type III secretion is bypassed by FlhDC upregulation. *Mol. Microbiol.* 75 376–393. 10.1111/j.1365-2958.2009.06973.x 19919668 PMC3194100

[B18] Fahrner K. A. Ryu W. S. Berg H. C. (2003). Biomechanics: bacterial flagellar switching under load. *Nature* 423:938. 10.1038/423938a 12827190

[B19] Ford K. M. Chawla R. Lele P. P. (2017). Biophysical characterization of flagellar motor functions. *J. Vis. Exp.* 119:e55240. 10.3791/55240 28190023 PMC5352267

[B20] Fukumura T. Makino F. Dietsche T. Kinoshita M. Kato T. Wagner S. (2017). Assembly and stoichiometry of the core structure of the bacterial flagellar type III export gate complex. *PLoS Biol.* 15:e2002281. 10.1371/journal.pbio.2002281 28771466 PMC5542437

[B21] Gillen K. L. Hughes K. T. (1991). Negative regulatory loci coupling flagellin synthesis to flagellar assembly in *Salmonella* typhimurium. *J. Bacteriol.* 173 2301–2310. 10.1128/jb.173.7.2301-2310.1991 1848842 PMC207783

[B22] Harshey R. M. (2003). Bacterial Motility on a surface: many ways to a common goal. *Annu. Rev. Microbiol.* 57 249–273. 10.1146/annurev.micro.57.030502.091014 14527279

[B23] Harshey R. M. Matsuyama T. (1994). Dimorphic transition in *Escherichia coli* and *Salmonella typhimurium*: surface-induced differentiation into hyperflagellate swarmer cells. *Proc. Natl. Acad. Sci. U.S.A.* 91 8631–8635. 10.1073/pnas.91.18.8631 8078935 PMC44660

[B24] Henrichsen J. (1972). Bacterial surface translocation: a survey and a classification. *Bacteriol. Rev.* 36 478–503. 4631369 10.1128/br.36.4.478-503.1972 PMC408329

[B25] Homma M. Kutsukake K. Hasebe M. Iino T. Macnab R. M. (1990). FlgB, FlgC, FlgF and FlgG: a family of structurally related proteins in the flagellar basal body of *Salmonella typhimurium*. *J. Mol. Biol.* 211 465–477. 10.1016/0022-2836(90)90365-S 2129540

[B26] Hughes A. C. Kearns D. B. (2017). “Swimming, swarming and sliding motility in bacillus subtilis. *Bacillus*,” in *Cellular and Molecular Biology*, 3 Edn, ed. Graumann P. L. (Poole: Caister Academic Press), 415–438. 10.21775/9781910190579-14

[B27] Kearns D. B. (2010). A field guide to bacterial swarming motility. *Nat. Rev. Microbiol.* 8 634–644. 10.1038/nrmicro2405 20694026 PMC3135019

[B28] Kim W. Killam T. Sood V. Surette M. G. (2003). Swarm-cell differentiation in *Salmonella*enterica Serovar Typhimurium results in elevated resistance to multiple antibiotics. *J. Bacteriol.* 185 3111–3117. 10.1128/JB.185.10.3111-3117.2003 12730171 PMC154059

[B29] Kim W. Surette M. G. (2003). Swarming populations of *Salmonella* represent a unique physiological state coupled to multiple mechanisms of antibiotic resistance. *Biol. Proc. Online* 5 189–196. 10.1251/bpo61 14615815 PMC248473

[B30] Kojima S. Blair D. F. (2004). The bacterial flagellar motor: structure and function of a complex molecular machine. *Int. Rev. Cytol.* 233 93–134. 10.1016/S0074-7696(04)33003-2 15037363

[B31] Konishi M. Kanbe M. McMurry J. L. Aizawa S. I. (2009). Flagellar formation in C-ring-defective mutants by overproduction of FliI, the atpase specific for flagellar type III secretion. *J. Bacteriol.* 191 6186–6191. 10.1128/JB.00601-09 19648242 PMC2747906

[B32] Kuchma S. L. Delalez N. J. Filkins L. M. Snavely E. A. Armitage J. P. O’Toole G. A. (2015). Cyclic di-GMP-mediated repression of swarming motility by *Pseudomonas aeruginosa* PA14 requires the MotAB stator. *J. Bacteriol.* 197 420–430. 10.1128/JB.02130-14 25349157 PMC4285984

[B33] Lauga E. DiLuzio W. R. Whitesides G. M. Stone H. A. (2006). Swimming in circles: motion of bacteria near solid boundaries. *Biophys. J.* 90 400–412. 10.1529/biophysj.105.069401 16239332 PMC1367047

[B34] Lele P. P. Berg H. C. (2015). Switching of bacterial flagellar motors triggered by mutant flig. *Biophys. J.* 108 1275–1280. 10.1016/j.bpj.2015.02.004 25762339 PMC4375538

[B35] Lele P. P. Branch R. W. Nathan V. S. J. Berg H. C. (2012). Mechanism for adaptive remodeling of the bacterial flagellar switch. *Proc. Natl. Acad. Sci. U.S.A.* 109 20018–20022. 10.1073/pnas.1212327109 23169659 PMC3523824

[B36] Lele P. P. Hosu B. G. Berg H. C. (2013). Dynamics of mechanosensing in the bacterial flagellar motor. *Proc. Natl. Acad. Sci. U.S.A.* 110 11839–11844. 10.1073/pnas.1305885110 23818629 PMC3718179

[B37] Lele P. P. Roland T. Shrivastava A. Chen Y. Berg H. C. (2015a). The flagellar motor of *Caulobacter crescentus* generates more torque when a cell swims backwards. *Nat. Phys.* 12 175–178. 27499800 10.1038/nphys3528 PMC4973516

[B38] Lele P. P. Shrivastava A. Roland T. Berg H. C. (2015b). Response thresholds in bacterial chemotaxis. *Science Advances* 1 e1500299. 10.1126/sciadv.1500299 26601280 PMC4646794

[B39] Li H. Sourjik V. (2011). Assembly and stability of flagellar motor in *Escherichia coli*. *Mol. Microbiol.* 80 886–899. 10.1111/j.1365-2958.2011.07557.x 21244534

[B40] Liarzi O. Barak R. Bronner V. Dines M. Sagi Y. Shainskaya A. (2010). Acetylation represses the binding of CheY to its target proteins. *Mol. Microbiol.* 76 932–943. 10.1111/j.1365-2958.2010.07148.x 20398208

[B41] Macnab R. M. (2003). How bacteria assemble flagella. *Annu. Rev. Microbiol.* 57 77–100. 10.1146/annurev.micro.57.030502.090832 12730325

[B42] Mariconda S. Wang Q. Harshey R. M. (2006). A mechanical role for the chemotaxis system in swarming motility. *Mol. Microbiol.* 60 1590–1602. 10.1111/j.1365-2958.2006.05208.x 16796690

[B43] McMurry J. L. Murphy J. W. González-Pedrajo B. (2006). The FliN-FliH interaction mediates localization of flagellar export ATPase FliI to the C ring complex. *Biochemistry* 45 11790–11798. 10.1021/bi0605890 17002279

[B44] Minamino T. (2018). Hierarchical protein export mechanism of the bacterial flagellar type III protein export apparatus. *FEMS Microbiol. Lett.* 365:fny117 10.1093/femsle/fny117 29850796

[B45] Mobley H. L. T. Belas R. (1995). Swarming and pathogenicity of *Proteus mirabilis* in the urinary tract. *Trends Microbiol.* 3 280–284. 10.1016/S0966-842X(00)88945-3 7551643

[B46] Overhage J. Bains M. Brazas M. D. Hancock R. E. (2008). Swarming of *Pseudomonas aeruginosa* is a complex adaptation leading to increased production of virulence factors and antibiotic resistance. *J. Bacteriol.* 190 2671–2679. 10.1128/JB.01659-07 18245294 PMC2293252

[B47] Pandini A. Morcos F. Khan S. (2016). The gearbox of the bacterial flagellar motor switch. *Structure* 24 1209–1220. 10.1016/j.str.2016.05.012 27345932 PMC4938800

[B48] Park S. Y. Lowder B. Bilwes A. M. Blair D. F. Crane B. R. (2006). Structure of FliM provides insight into assembly of the switch complex in the bacterial flagella motor. *Proc. Natl. Acad. Sci. U.S.A.* 103 11886–11891. 10.1073/pnas.0602811103 16882724 PMC1567671

[B49] Paul K. Brunstetter D. Titen S. Blair D. F. (2011a). A molecular mechanism of direction switching in the flagellar motor of *Escherichia coli*. *Proc. Natl. Acad. Sci. U.S.A.* 108 17171–17176. 10.1073/pnas.1110111108 21969567 PMC3193218

[B50] Paul K. Gonzalez-Bonet G. Bilwes A. M. Crane B. R. Blair D. (2011b). Architecture of the flagellar rotor. *EMBO J.* 30 2962–2971. 10.1038/emboj.2011.188 21673656 PMC3160249

[B51] Paul K. Harmon J. G. Blair D. F. (2006). Mutational Analysis of the Flagellar Rotor Protein FliN: Identification of Surfaces Important for Flagellar Assembly and Switching. *J. Bacteriol.* 188 5240–5248. 10.1128/JB.00110-06 16816196 PMC1539977

[B52] Ryu W. S. Berry R. M. Berg H. C. (2000). Torque-generating units of the flagellar motor of *Escherichia coli* have a high duty ratio. *Nature* 403 444–447. 10.1038/35000233 10667798

[B53] Sarkar M. K. Paul K. Blair D. (2010). Chemotaxis signaling protein CheY binds to the rotor protein FliN to control the direction of flagellar rotation in *Escherichia coli*. *Proc. Natl. Acad. Sci. U.S.A.* 107 9370–9375. 10.1073/pnas.1000935107 20439729 PMC2889077

[B54] Scharf B. E. Fahrner K. A. Turner L. Berg H. C. (1998). Control of direction of flagellar rotation in bacterial?chemotaxis. *Proc. Natl. Acad. Sci. U.S.A.* 95 201–206. 10.1073/pnas.95.1.201 9419353 PMC18175

[B55] Schuster S. C. Khan S. (1994). The bacterial flagellar motor. *Annu. Rev. Biophys. Biomol. Struct.* 23 509–539. 10.1146/annurev.bb.23.060194.002453 7919791

[B56] Sourjik V. Berg H. C. (2000). Localization of components of the chemotaxis machinery of *Escherichia coli* using fluorescent protein fusions. *Mol. Microbiol.* 37 740–751. 10.1046/j.1365-2958.2000.02044.x 10972797

[B57] Sourjik V. Berg H. C. (2002). Binding of the *Escherichia coli* response regulator CheY to its target measured *in vivo* by fluorescence resonance energy transfer. *Proc. Natl. Acad. Sci. U.S.A.* 99 12669–12674. 10.1073/pnas.192463199 12232047 PMC130518

[B58] Tang H. Blair D. F. (1995). Regulated underexpression of the FliM protein of *Escherichia coli* and evidence for a location in the flagellar motor distinct from the MotA/MotB torque generators. *J. Bacteriol.* 177 3485–3495. 10.1128/jb.177.12.3485-3495.1995 7768858 PMC177053

[B59] Terahara N. Noguchi Y. Nakamura S. Kami-ike N. Ito M. Namba K. (2017). Load- and polysaccharide-dependent activation of the Na+-type MotPS stator in the Bacillus subtilis flagellar motor. *Sci. Rep.* 7:46081. 10.1038/srep46081 28378843 PMC5380961

[B60] Tipping M. J. Delalez N. J. Lim R. Berry R. M. Armitage J. P. (2013). Load-dependent assembly of the bacterial flagellar motor. *mBio* 4:e00551-13. 10.1128/mBio.00551-13 23963182 PMC3747592

[B61] Togashi F. Yamaguchi S. Kihara M. Aizawa S. I. Macnab R. M. (1997). An extreme clockwise switch bias mutation in fliG of *Salmonella typhimurium* and its suppression by slow-motile mutations in motA and motB. *J. Bacteriol.* 179 2994–3003. 10.1128/jb.179.9.2994-3003.1997 9139919 PMC179065

[B62] Turner L. Ping L. Neubauer M. Berg H. C. (2016). Visualizing flagella while tracking bacteria. *Biophys. J.* 111 630–639. 10.1016/j.bpj.2016.05.053 27508446 PMC4982932

[B63] Tuson H. H. Copeland M. F. Carey S. Sacotte R. Weibel D. B. (2013). Flagellum density regulates *Proteus mirabilis* swarmer cell motility in viscous environments. *J. Bacteriol.* 195 368–377. 10.1128/JB.01537-12 23144253 PMC3553826

[B64] Wang Q. Frye J. G. McClelland M. Harshey R. M. (2004). Gene expression patterns during swarming in *Salmonella typhimurium*: genes specific to surface growth and putative new motility and pathogenicity genes. *Mol. Microbiol.* 52 169–187. 10.1111/j.1365-2958.2003.03977.x 15049819

[B65] Wang Q. Suzuki A. Mariconda S. Porwollik S. Harshey R. M. (2005). Sensing wetness: a new role for the bacterial flagellum. *EMBO J.* 24 2034–2042. 15889148 10.1038/sj.emboj.7600668 PMC1142604

[B66] Yang A. Tang W. S. Si T. Tang J. X. (2017). Influence of physical effects on the swarming motility of *Pseudomonas aeruginosa*. *Biophys. J.* 112 1462–1471. 10.1016/j.bpj.2017.02.019 28402888 PMC5389960

[B67] Yuan J. Fahrner K. A. Turner L. Berg H. C. (2010). Asymmetry in the clockwise and counterclockwise rotation of the bacterial flagellar motor. *Proc Natl Acad Sci U.S.A.* 107 12846–12849. 10.1073/pnas.1007333107 20615986 PMC2919929

[B68] Zhang H. P. Be’er A. Smith R. S. Florin E.-L. Swinney H. L. (2009). Swarming dynamics in bacterial colonies. *Europhys. Lett.* 87:48011 10.1209/0295-5075/87/48011

[B69] Zhao R. Pathak N. Jaffe H. Reese T. S. Khan S. (1996). FliN is a major structural protein of the C-ring in the *Salmonella typhimurium* flagellar basal body. *J. Mol. Biol.* 261 195–208. 10.1006/jmbi.1996.0452 8757287

