# Supplementary material for: Switching and Torque Generation in Swarming E. coli
Source: Front Microbiol. 2018 Sep 18;9:2197. doi: 10.3389/fmicb.2018.02197 (PMC6153309; doi:10.3389/fmicb.2018.02197)
Supplement: Supplementary file 1 [file Data_Sheet_1.docx]

**Supplementary Information**

**
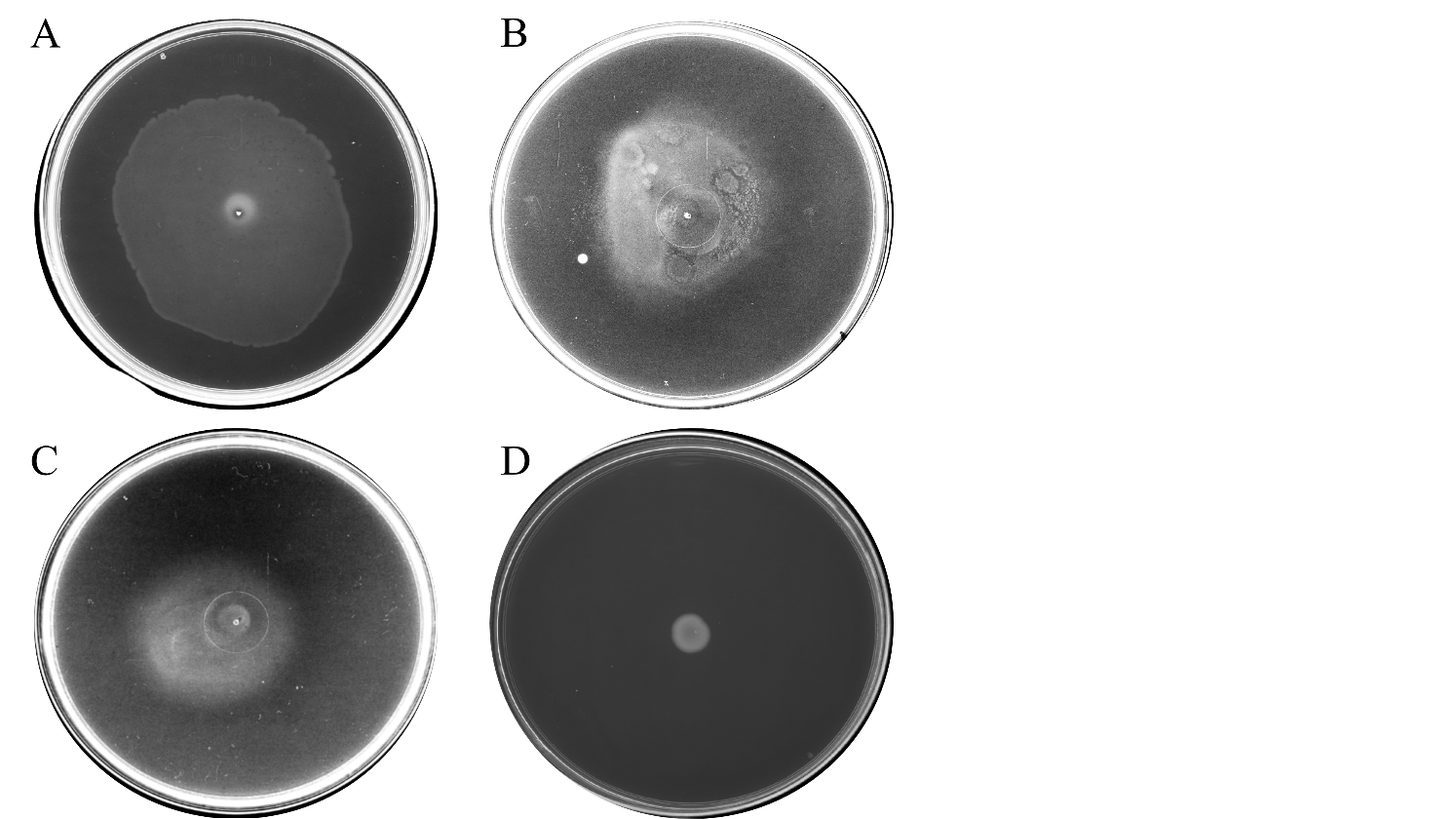
**

Figure S1. Representative swarm assays for the strains of interest. (A) A standard swarm assay for the wildtype strain; (B) Swarming in CCW strain on agar surfaces that have additional moisture; (C) Swarming in CW strain on agar surfaces that have additional moisture; (D) Inability of the *fliG^CW^* strain to swarm despite the addition of moisture to the agar surface.

| Target gene | Primer set | Sequence (5’ to 3’) | Product size (bp) |
| --- | --- | --- | --- |
| *gapA* | GapA_RT_Fwd  GapA_RT_Rev | AAGTTGGTGTTGACGTTGTCGCTG  ATAACCACTTTCTTCGCACCAGCGG | 97 |
| *fliC* | FliC_RT_Fwd  FliC_RT_Rev | ACAGCCTCTCGCTGATCACTCAAA  GCGCTGTTAATACGCAAGCCAGAA | 100 |

**Table S1.** Primer information for qPCR studies.

| Strain | ΔΔCt |
| --- | --- |
| Wildtype | 2.01 |
| CCW | 0.77 |
| FliG^CW^ | 0.93 |

Table S2. qPCR results for agar-grown strains. ΔΔCt values > 2 were considered significant.


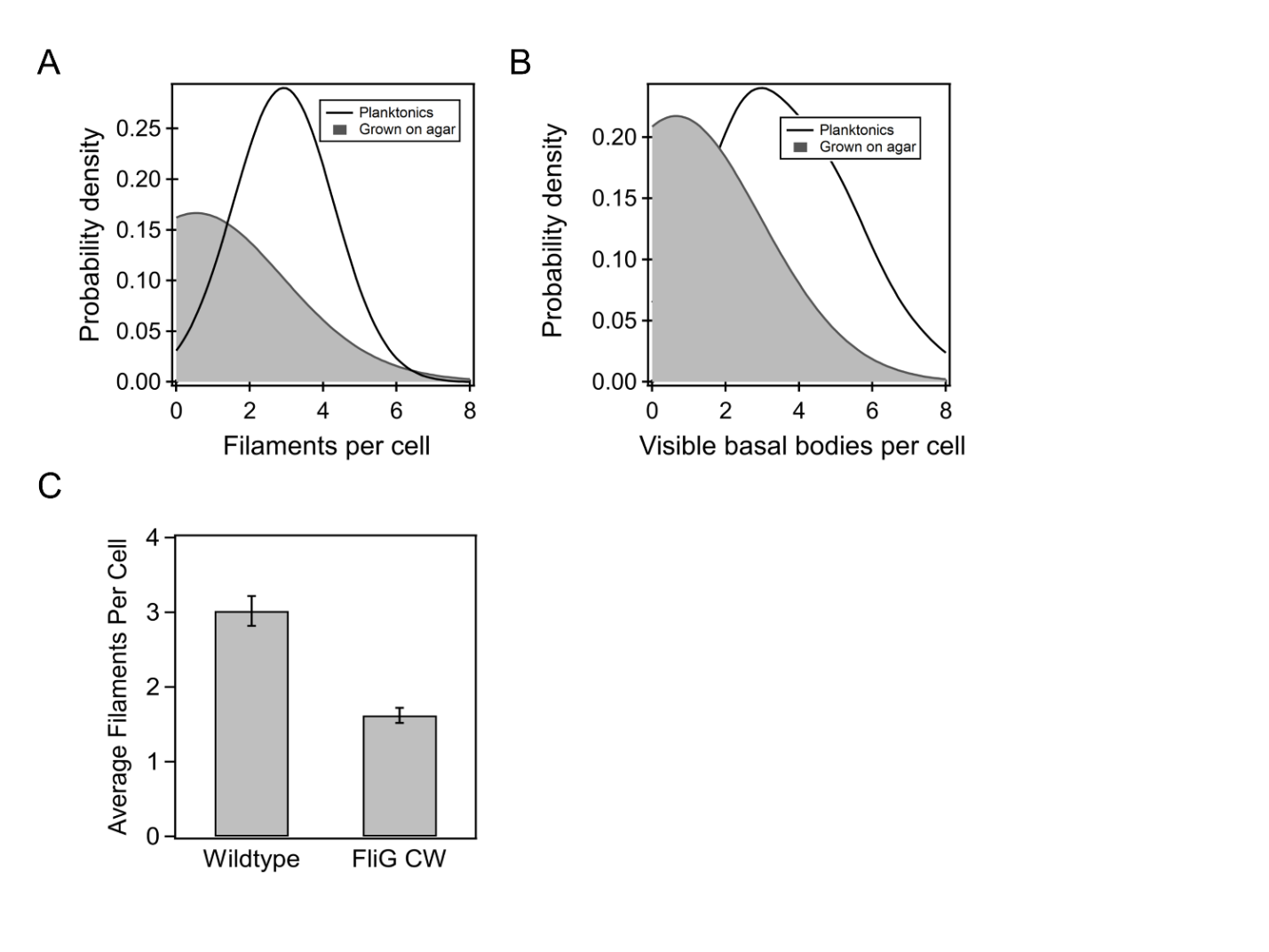


**Figure S2.** Filament counts via fluorescence-visualization in wildtype swarmer cells and FliG^CW^ cells grown on agar surfaces.


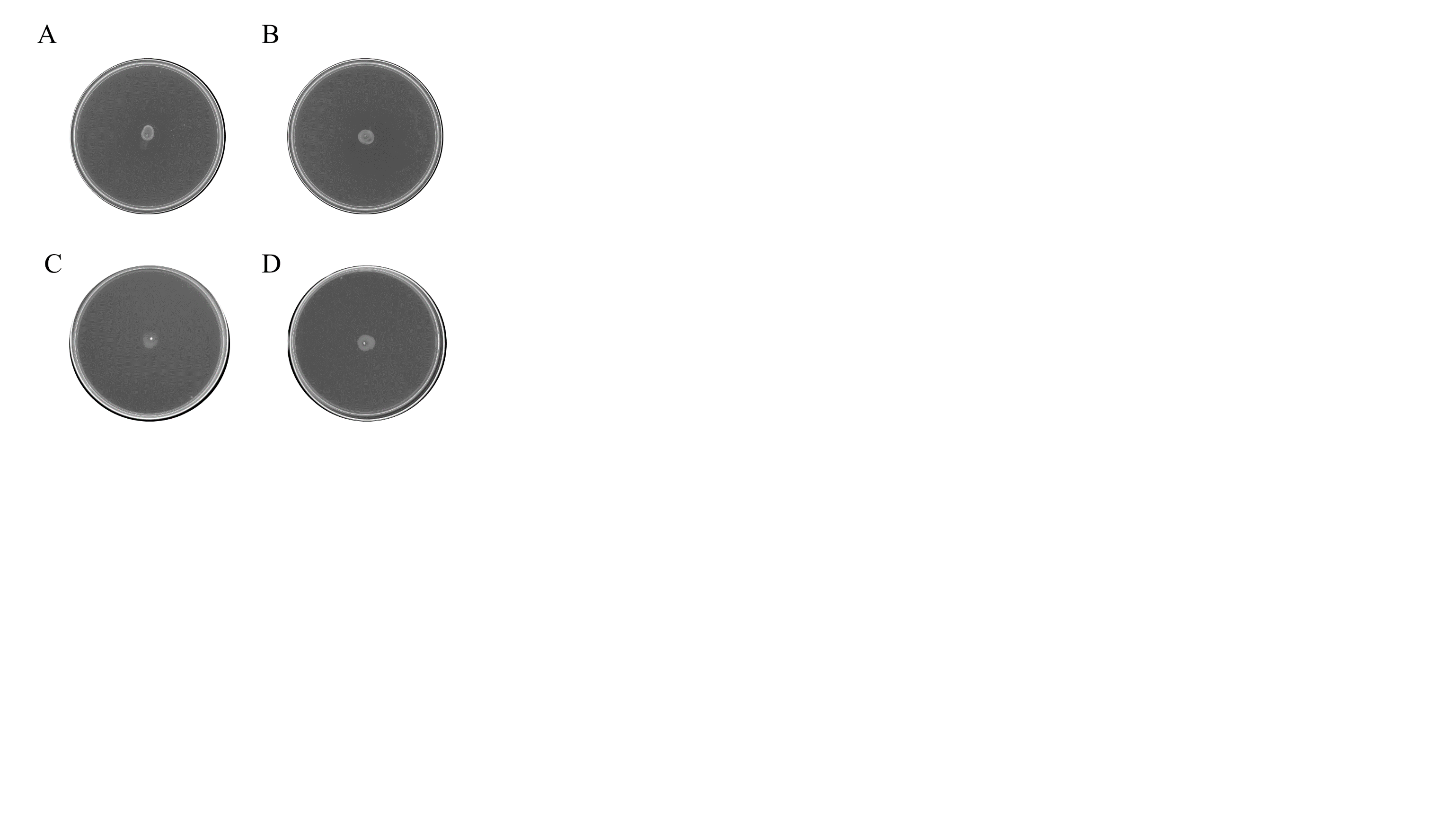


**Figure S3.** Representative swarms from overexpression assays. A) FliI overexpression in FliG^CW^ B) FlhA overexpression in FliG^CW^

*
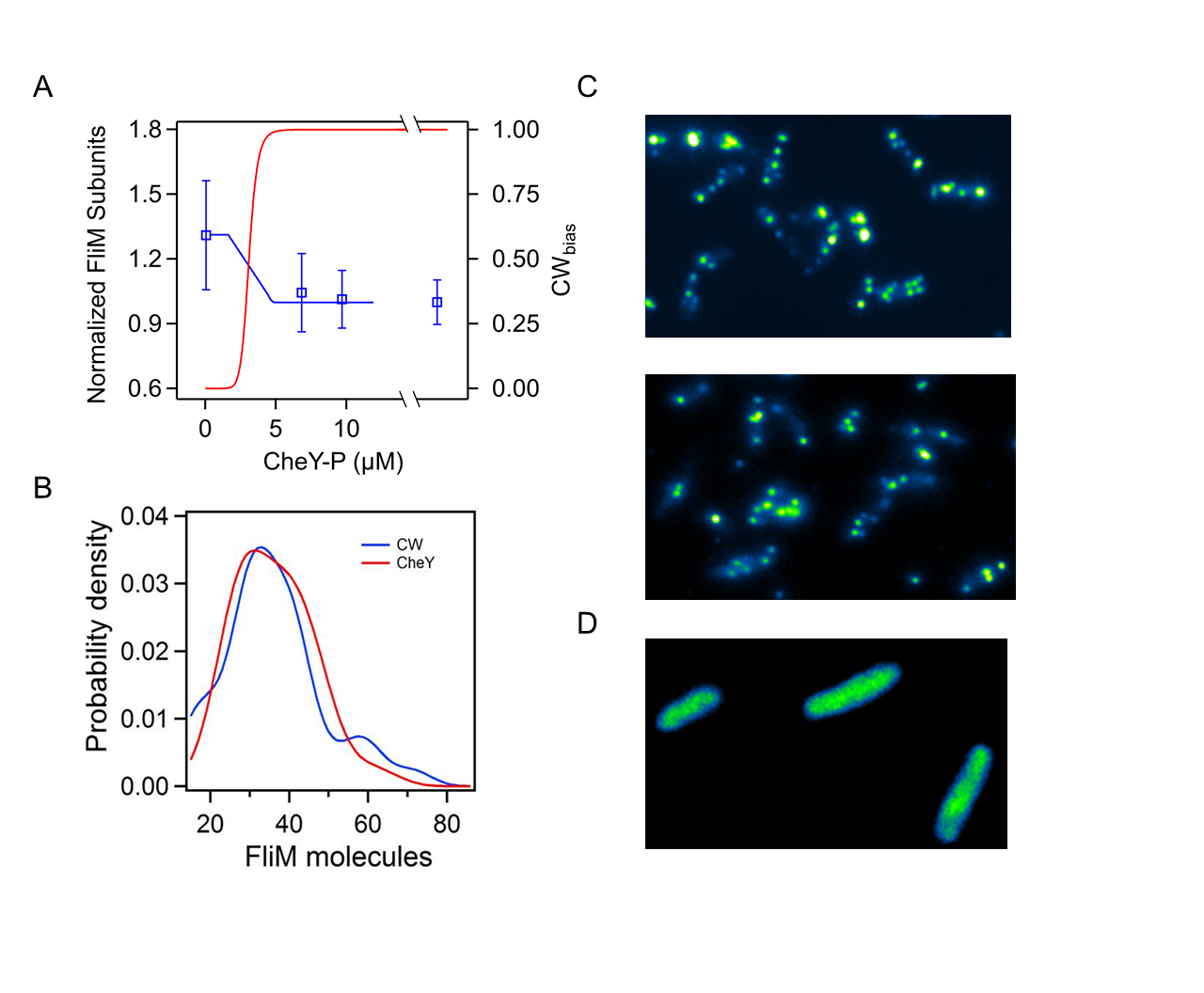
*

**Figure S4**. **A)** Dependence of normalized FliM numbers on the amount of intracellular CheY-P and CW_bias_  (Lele *et al*., 2012) There are fewer FliM molecules in CW-rotating motors and more FliM molecules in CCW-rotating motors. **B)** Comparisons between distributions of FliM molecules in rotating-flagellar motors in the FliG^CW^ (blue) and a CW-only strain that carried the wildtype *fliG* allele and an excess pool of phosphorylated CheY (red). **C)** Representative images of putative flagellar preassemblies in cells belonging to a strain that carried the native *fliG* allele (left) and the *fliG^CW^* allele (right). The assemblies are indicated by localization of fluorescently-labeled FliM subunits. **D)** Representative image of a strain that lacks FliG and carries fluorescently-labeled FliM. As is clear, there is a complete lack of localization.

The FliG^CW^ mutant employed in this work has been previously characterized in *E. coli* with the aid of biophysical assays (Lele *et al.,* 2012, Shrivastava *et al.* 2015, Lele and Berg, 2015). Figure S3 indicates a subset of the data obtained via total internal reflection fluorescence microscopy (TIRF). Fig S3A indicates the dependence of the average number of FliM molecules in tethered motors as a function of cytoplasmic CheY-P levels (Figure 1, Lele *et al*., PNAS 2012). The last data point represents the mean numbers in a strain carrying the *fliG^CW^* allele. In Figure S3B, the kernel density estimates are shown for the number of FliM molecules in two types of motors: FliG^CW^ motors (blue) and wildtype motors (FliG^WT^) that are forced to rotate CW-only due to an excess pool of CheY-P molecules (Δ*cheRcheBcheZ*, *cheY-ptrc99A*). The difference in means was not statistically-significant at a 0.01 level. Figure S3C shows the similarities between localization of FliM molecules in the cell bodies in strains carrying the wildtype *fliG* (left) and the *fliG^CW^* alleles on the genome. Together with the torque data in Figure 2, these results indicate that the assembly of FliM is not affected by the presence of the *fliG^CW^* mutation, per se, although the direction of motor rotation certainly plays a role (Lele *et al*., PNAS 2012). Figure S4D indicates how cells that are unable to form a C-ring appear when FliM fusions are visualized via TIRF. Typically, the cells appear uniformly bright with no localization of the fluorescent signals.

**
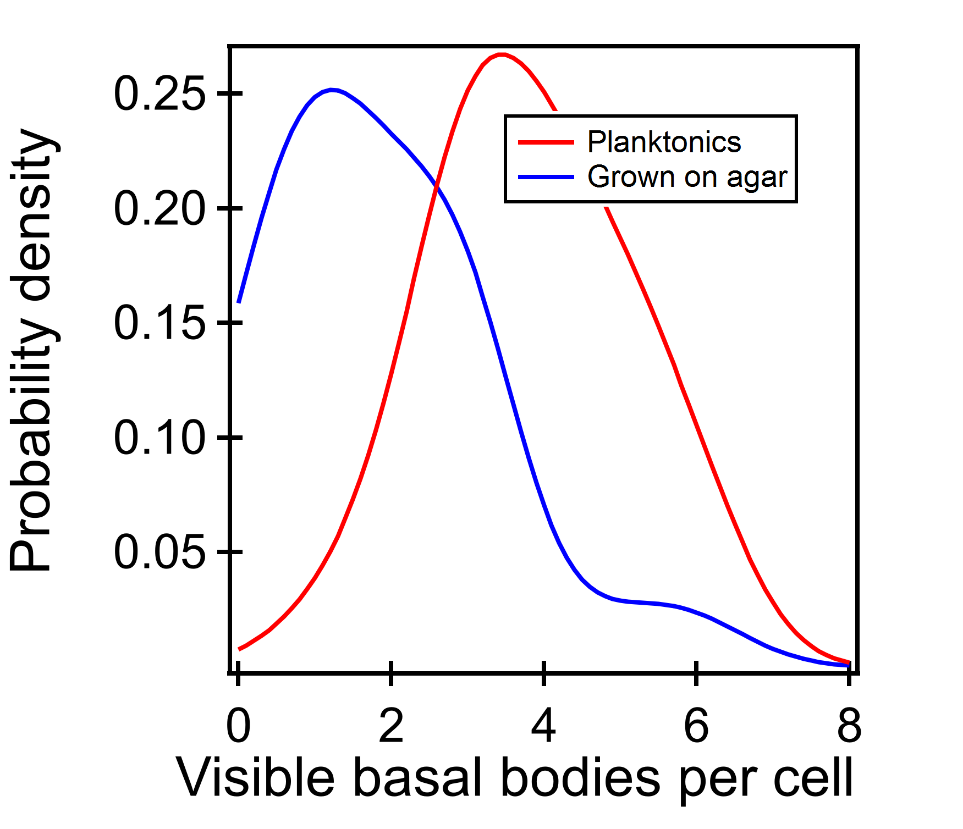
**

**Figure S5**. Relative number of flagellar preassemblies in the planktonic and agar-grown cells in a strain that carried the native *fliG* and *fliM-eYFP-fliM* alleles and a constitutively-active form of CheY (CheYD13KY106W) on an inducible plasmid. The number of putative flagellar preassemblies were determined from TIRF measurements. There were fewer preassemblies in the agar-grown cells (1.84 ± 0.25 per cell, n = 31) relative to the planktonic cells (3.83 ± 0.21 per cell, n = 36). The difference in the means was significant (p < 0.05).
